# Supplementary figures and images for: Objective crystallographic symmetry classifications of a noisy crystal pattern with strong Fedorov-type pseudo­symmetries and its optimal image-quality enhancement
Source: Acta Crystallogr A Found Adv. 2022 Apr 28;78(Pt 3):172–99. doi: 10.1107/S2053273322000845 (PMC9062829; doi:10.1107/S2053273322000845)

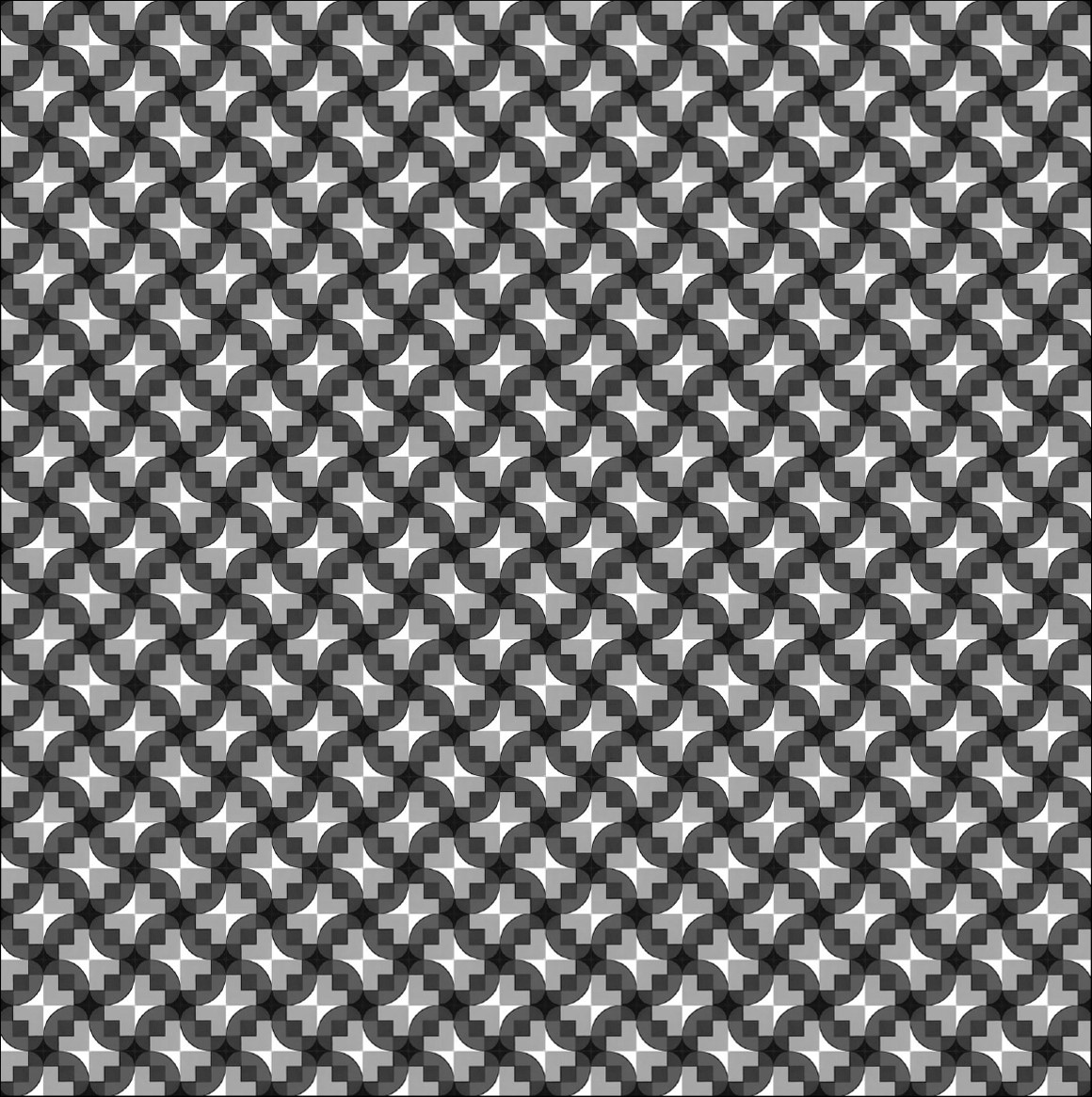

Supplement: Supplementary file 1 [file a-78-00172-sup1.tif]

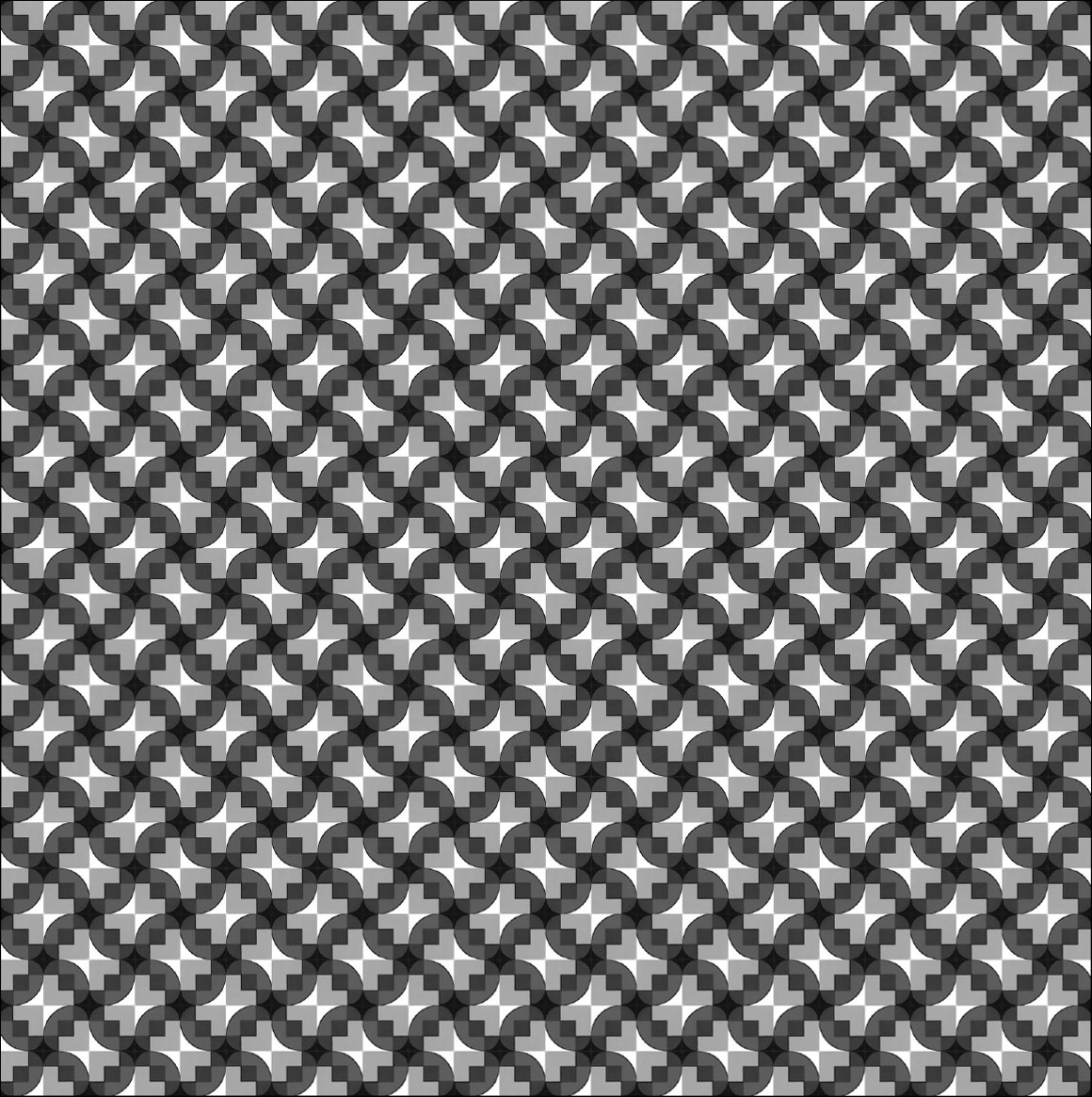

Supplement: Supplementary file 2 [file a-78-00172-sup2.jpg]
